# P-509. Impact of an Innovative Educational Initiative Using Community-Based Organization Partnerships to Reach Young Adults at Risk for HIV

**DOI:** 10.1093/ofid/ofae631.708

**Published:** 2025-01-29

**Authors:** Meredith E Clement, Leah Molloy, Laura Simone, Chris Napolitan, Jeffrey D Carter, Kelly E Pillinger

**Affiliations:** Louisiana State University Health Science Center–New Orleans, New Orleans, LA; PRIME Education, Brighton, Michigan; PRIME Education, LLC, Fort Lauderdale, Florida; PRIME Education, Brighton, Michigan; PRIME Education, LLC, Fort Lauderdale, Florida; PRIME Education, Brighton, Michigan

## Abstract

**Background:**

Young adults (YA) are particularly vulnerable to acquiring HIV due to low self-perceived risk, low rates of HIV testing, and lack of knowledge/awareness about pre-exposure prophylaxis (PrEP).

**Figure:**
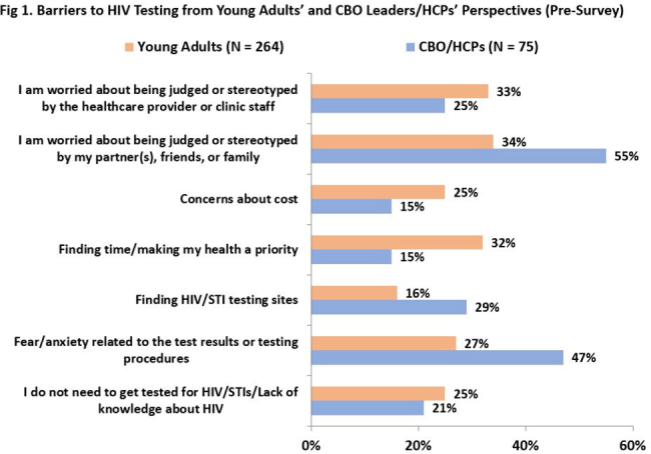

**Methods:**

Between 3/23 – 8/23, six education sessions for YAs were conducted in the Southeastern U.S. by local healthcare professionals (HCPs) along with community-based organizations (CBOs) in non-clinical settings (e.g. college campuses, breweries). Pre- and post-surveys were administered, with follow-up surveys 3 weeks later. A scale-up education toolkit was disseminated to HCPs nationwide.

**Results:**

A total of 264 YAs participated in the education sessions (mean age: 24 years; 85% female; 54% Caucasian; 87% heterosexual) with 29% previously tested for HIV and 21% who previously discussed PrEP with a HCP. YA’s knowledge of different forms of PrEP improved significantly (38% pre- vs. 96% post-activity; p < 0.001) as did their confidence in protecting themselves from acquiring HIV (55% pre vs. 93% post; p < 0.001). In addition, awareness of local resources for HIV testing improved from 60% to 93% (p < 0.001) with a significant increase in YAs who stated they were likely to get tested for HIV (26% pre vs. 63% post, p < 0.001).

Seventy-five HCPs and CBO leaders also participated. Interestingly, there was a disconnect between CBO/HCPs’ perceptions of barriers to HIV/STI testing compared to YAs’ reported challenges (Fig 1). All providers stated they were likely/very likely to organize another session. A follow-up survey of HCPs (N = 10) found they helped 58 YAs access HIV testing and 33 access PrEP since the education, with 70% of HCPs using the materials during 1:1 counseling with YAs, suggesting sustainability of the resources.

HCPs (N = 867) who reviewed the toolkit had improvements in their confidence in differentiating between PrEP options and identifying who would benefit from each approach (p < 0.001) and counseling YAs about PrEP (p < 0.001).

**Conclusion:**

Providing HIV prevention education to YAs in informal, community settings increases reach to those who may not have these discussions with their HCPs during regular visits. This type of educational initiative, which includes partnering with CBOs, is scalable and sustainable.

**Disclosures:**

**Meredith E. Clement, MD**, Gilead Sciences: Grant/Research Support|Viiv Pharmaceuticals: Advisor/Consultant **Kelly E. Pillinger, PharmD**, AHFS: Contractor

